# Draft Genome Sequence of the Free-Living, Iridescent Bacterium Tenacibaculum mesophilum Strain ECR

**DOI:** 10.1128/MRA.01302-20

**Published:** 2021-01-07

**Authors:** Rebecca L. Mickol, Artemis S. Louyakis, H. Lynn Kee, Lisa K. Johnson, Scott C. Dawson, Katherine R. Hargreaves, Grayson L. Chadwick, Dianne K. Newman, Jared R. Leadbetter, C. Titus Brown

**Affiliations:** a Arkansas Center for Space and Planetary Sciences, University of Arkansas, Fayetteville, Arkansas, USA; b American Society for Engineering Education, Washington, DC, USA; c Department of Molecular and Cell Biology, University of Connecticut, Storrs, Connecticut, USA; d Department of Biology, Stetson University, Deland, Florida, USA; e Department of Population Health and Reproduction, School of Veterinary Medicine, University of California Davis, Davis, California, USA; f Department of Microbiology and Molecular Genetics, College of Biological Science, University of California Davis, Davis, California, USA; g Department of Microbiology, The Ohio State University, Columbus, Ohio, USA; h Division of Geological and Planetary Sciences, California Institute of Technology, Pasadena, California, USA; i Division of Biology and Biological Engineering, California Institute of Technology, Pasadena, California, USA; j Division of Engineering and Applied Science, California Institute of Technology, Pasadena, California, USA; University of Delaware

## Abstract

Here, we report the genome sequence of Tenacibaculum mesophilum strain ECR, which was isolated from the river/ocean interface at Trunk River in Falmouth, Massachusetts. The isolation and sequencing were performed as part of the 2016 and 2018 Microbial Diversity courses at the Marine Biological Laboratory in Woods Hole, Massachusetts.

## Abstract (precis)

Here, we report the genome sequence of Tenacibaculum mesophilum strain ECR, which was isolated from the river/ocean interface at Trunk River, Falmouth, Massachusetts, USA. The isolation and sequencing were performed as part of the 2016 and 2018 Microbial Diversity courses at the Marine Biological Laboratory in Woods Hole, Massachusetts, USA.

## ANNOUNCEMENT

**FIG 1:**
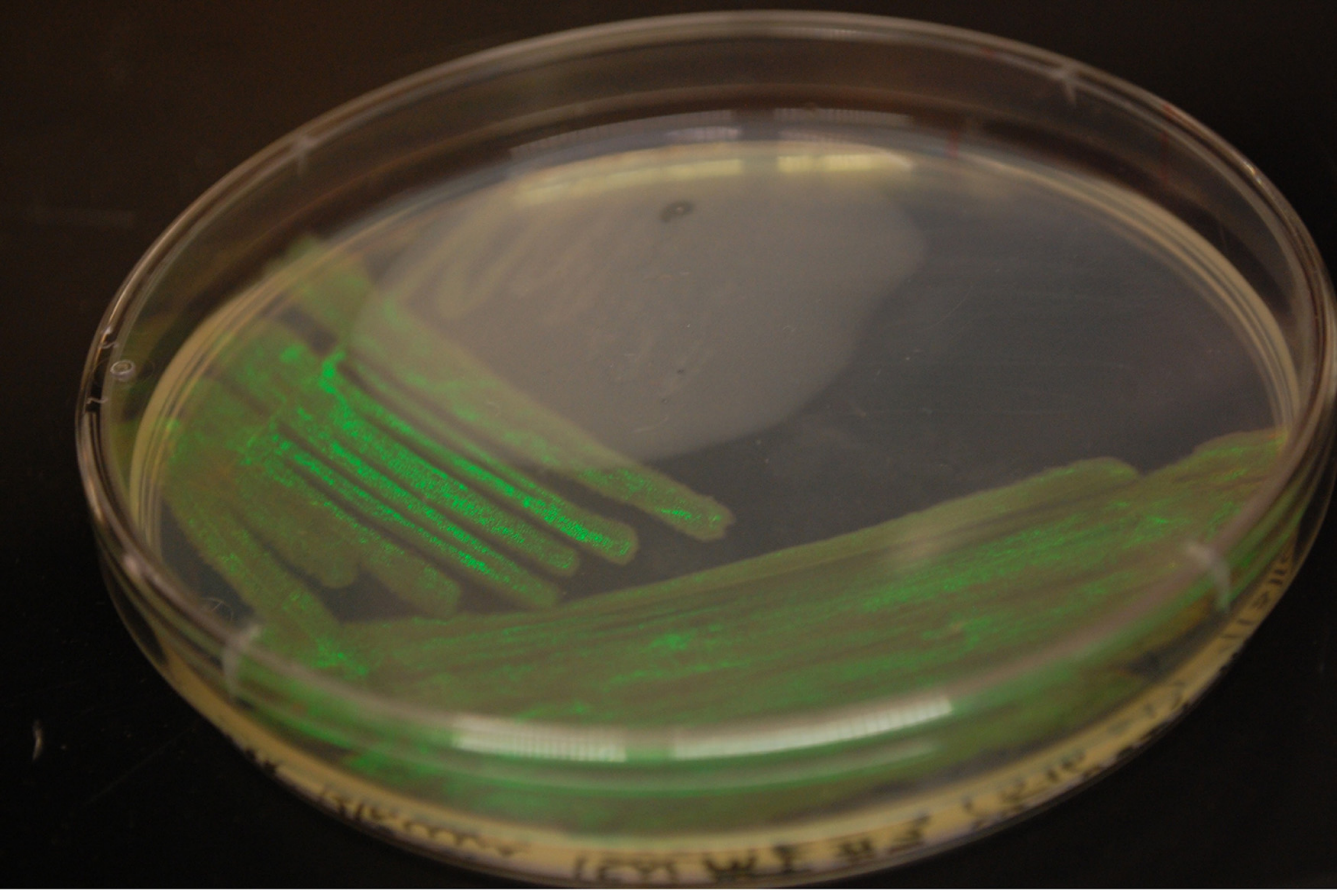
Streak plate illustrating vivid green fluorescence exhibited by Tenacibaculum mesophilum strain ECR.

A bacterial strain of Tenacibaculum mesophilum, designated strain ECR, was isolated from seawater from the river/ocean interface between Trunk River and the Atlantic Ocean in Falmouth, Massachusetts (41.524377N, −70.641422) in July 2016. Many *Tenacibaculum* species are important marine fish pathogens causing tenacibaculosis (1–5) and are often isolated from ulcers or lesions on diseased fish. A striking colony type from a free-living isolate, exhibiting vivid green iridescence (Fig. 1), was observed during the examination of a quantitative spread plate dilution series as described by Kee et al. (6). This genome sequence will enable further investigation into tenacibaculosis in fish (1–5), the mechanisms enabling vivid iridescence during colony formation, and gliding motility (7, 8).

This genome was sequenced using the Oxford Nanopore Technologies MinION Mk1B system using three R9.0 flow cells (FLO-MIN104) and two Illumina sequencing runs. For the first Illumina run and the MinION sequencing, a culture was grown overnight at 30°C in 500 ml seawater complete (SWC) broth. SWC medium consists of 1 liter of seawater base (containing, in 20 liters, 400 g NaCl, 60 g MgCl_2_·6H_2_O, 3 g CaCl_2_·2H_2_O, and 10 g KCl) supplemented with 5 g tryptone, 1 g yeast extract, and 3 ml glycerol. DNA was extracted using the Qiagen Genomic-tip 500/G kit and quantified using the QuantiFluor ONE double-stranded DNA (dsDNA) kit (Promega, Madison, WI, USA) with the Quantus system (Promega). MinION libraries were created using the standard 2D library preparation kit (SQK-NSK007). DNA for the first Illumina run was sheared on a sonicator (Covaris, Woburn, MA, USA) to 500 bp, and libraries were generated using the Ovation low-input kit (NuGen, Redwood City, CA, USA). Sequencing was conducted using the Illumina MiSeq system (2 × 300 bp). For the second Illumina run, an overnight culture was streaked onto an SWC agar plate at 30°C from a cryostock previously made in SWC broth with 25% glycerol and stored at −80°C. Prior to DNA extraction, a colony was picked and a culture was grown overnight at 30°C in SWC broth. DNA was extracted using the Maxwell RSC PureFood genetically modified organism (GMO) and authentication kit (Promega) and quantified using the QuantiFluor ONE dsDNA kit (Promega) with the Quantus system (Promega). Libraries were generated using the Nextera DNA Flex library kit (Illumina, San Diego, CA, USA). Genome sequencing was conducted using the Illumina HiSeq 2500 system (2 × 250 bp).

The total number of Illumina reads generated was 1,202,740, giving an estimated coverage of 90×. The MinION sequencing resulted in 2,112 reads (*N*_50_, 5,623 bp) assembled into two contigs, which were subsequently polished with the Illumina sequencing reads (trimmed with Sickle v1.33 [9]) using the Unicycler v0.4.7 (10) pipeline (https://github.com/alouyakis/TmesophilumECR). Default parameters were used for all software unless otherwise specified. All software versions are available on the GitHub page. The draft genome consists of two contigs with a total length of 3,405,417 bp, the estimated size of the genome, indicating that these are two fragments of a single chromosome and that this genome likely does not contain any plasmids. The G+C contents of the contigs were 31.87% and 31.58%. Genome completeness was estimated to be 100% (0.32% contamination) at the family level (*Flavobacteriaceae*) using CheckM v1.0.7 (11) and 92.67% (4.27% contamination) at the genus level. Prodigal v2.6.3 was used to identify open reading frames and resulted in 3,088 protein-coding genes.

### Data availability.

The GenBank accession number for this genome sequence is JAAQOW000000000 under NCBI BioProject PRJNA488075 (accession numbers SRR11313510, SRR11313511, and SRR11313512).
